# Small bowel obstruction secondary to impacted pancreatic necrosum through a spontaneous duodenal fistula: a first in the literature

**DOI:** 10.1093/jscr/rjae739

**Published:** 2024-11-24

**Authors:** Dora Laczko, Stephanie Skanes, David Hocking, David Peck, Jeffrey D Hawel, Richard Hilsden

**Affiliations:** Department of Surgery, Western University, London Health Sciences Centre, Victoria Hospital, 800 Commissioners Rd., Rm E4-112 London, ON N6A 5W9, Canada; Department of Surgery, Western University, London Health Sciences Centre, Victoria Hospital, 800 Commissioners Rd., Rm E4-112 London, ON N6A 5W9, Canada; Department of Medical Imaging, Western University, London Health Sciences Centre, Victoria Hospital, 800 Commissioners Rd., Rm E4-112 London, ON N6A 5W9, Canada; Department of Medical Imaging, Western University, London Health Sciences Centre, Victoria Hospital, 800 Commissioners Rd., Rm E4-112 London, ON N6A 5W9, Canada; Department of Surgery, Western University, London Health Sciences Centre, Victoria Hospital, 800 Commissioners Rd., Rm E4-112 London, ON N6A 5W9, Canada; Department of Surgery, Western University, London Health Sciences Centre, Victoria Hospital, 800 Commissioners Rd., Rm E4-112 London, ON N6A 5W9, Canada

**Keywords:** general surgery, diagnostic imaging, pancreas, pancreatitis, bowel obstruction, bezoar, endoscopy

## Abstract

A 58-year-old man presented with alcohol-induced acute pancreatitis. Imaging revealed complete necrosis of the pancreatic parenchyma. After initial non-operative management, the patient developed a duodenal ulcer and an upper gastrointestinal bleed and later spontaneously formed a fistula between the pancreas and the first segment of the duodenum. Through this fistula, the entire pancreatic necrosum migrated to the distal small bowel, where it became entrapped as a bezoar, causing a high-grade small bowel obstruction. The patient then proceeded to laparotomy, where the obstruction was resolved by removing the necrosum through an enterotomy. No surgical intervention to the pancreatic bed or duodenum was required, and he was ultimately discharged home on insulin and pancreatic enzymes. This is the first reported case in the medical literature of a spontaneous duodenal fistula leading to small bowel obstruction due to a bezoar of pancreatic necrosum.

## Introduction

Pancreatitis is a common cause of emergency department presentation and admission to the hospital [1]. A feared complication of this disease is pancreatic necrosis, carrying a high mortality rate [2]. Over time, the management of this condition has evolved from open surgical approaches to less invasive surgical and endoscopic strategies. The endoscopic approach involves placing a stent through the stomach into the cavity containing the pancreatic necrosum, resulting in a fistula between the pancreatic pseudocyst and the stomach [3]. The case presented in this article is a first-time report of a bowel obstruction secondary to the migration of a pancreatic necrosum through a spontaneous pancreaticoduodenal fistula.

## Case report

We present the case of a 58-year-old gentleman with a past medical history of coronary artery disease, obstructive sleep apnea, hyperlipidemia, and hypertension. He initially presented to a large community hospital in April 2022 with clinical, biochemical, and radiographic evidence of acute, uncomplicated pancreatitis. The patient reported a history of significant alcohol use, leading to a diagnosis of acute alcohol-induced pancreatitis. He recovered with supportive management and was discharged home in early May.

Three weeks later, he returned to the emergency department with diffuse abdominal pain, similar to his previous pancreatitis. Repeat imaging revealed complete necrosis of the pancreatic parenchyma. At this point, the patient was transferred from the community hospital to a tertiary hospital with expertise in complex pancreatic conditions. While at the tertiary care facility, he clinically improved and was again discharged home from the hospital.

Two weeks following the second discharge, the patient presented to the tertiary care hospital’s emergency department with worsening abdominal pain and dyspnea. Computed tomography (CT) imaging showed an increase in the size of the necrotic pancreatic collection. Interventional radiology attempted to insert a transgastric drain into the collection, but it was deemed to be a solid immature phlegmon, and more time was needed before another attempt at drain insertion would be appropriate. In early June, the patient developed an upper GI bleed, and an esophagogastroduodenoscopy revealed a large duodenal ulcer with an overlying clot, which was injected with epinephrine. The patient showed clinical improvement following this, and repeat imaging showed a stable appearance of the pancreas. He was discharged home in early July.

The patient’s final presentation to the hospital occurred in mid-July. A CT scan on admission showed a similar-appearing pancreas but with a new fistula between the pancreas and the first segment of the duodenum. Given this new connection, we consulted a surgical endoscopist for consideration of debridement of the collection through the fistula itself.

Endoscopic findings during the procedure revealed a surprisingly full stomach and distended duodenum, despite the patient’s nil per os (NPO) status. The first part of the duodenum demonstrated a large fistula tract had spontaneously formed with the pancreatic bed. This cavity was deep, extending all the way to the pancreatic tail. It was surprisingly empty, and the previously identified phlegmonous material from the CT scan was not found during the endoscopic procedure.

Unfortunately, the patient became systemically unwell following the procedure, requiring transfer to the intensive care unit. An emergent CT scan was performed to assess possible complications and revealed a mechanical small bowel obstruction with an impacted tissue bezoar at the ileocecal value. Vomiting from this obstruction had also resulted in an aspiration pneumonia. The patient’s respiratory status improved over the following 24 h, and he was brought to the operating room emergently to relieve the obstruction.

**Figure 1:**
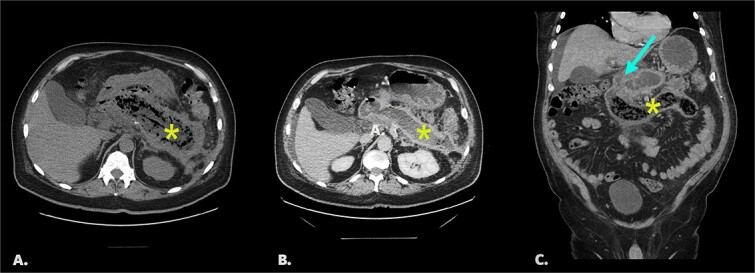
(A) Initial non-contrast CT. Axial image shows extensive pancreatic necrosis and phlegmon (*). 1B/C Follow up CT with IV contrast (portal venous phase). Image 1B: axial image shows fluid replacement of tissue in the pancreatic bed (*). Image IC: coronal image shows fistula between pancreatic bed and duodenum (arrow) and bezoar in the distal duodenum (*).

On initial CT imaging, avascular phlegmon and necrotic pancreatic tissue were observed in the expected retroperitoneal location of the pancreas (Fig. 1A).

On follow-up CT imaging, the pancreatic bed was fluid filled with tubular, avascular mottled material in the distal duodenum, representing pancreatic bezoar (Fig. 1B and 1C).

**Figure 2:**
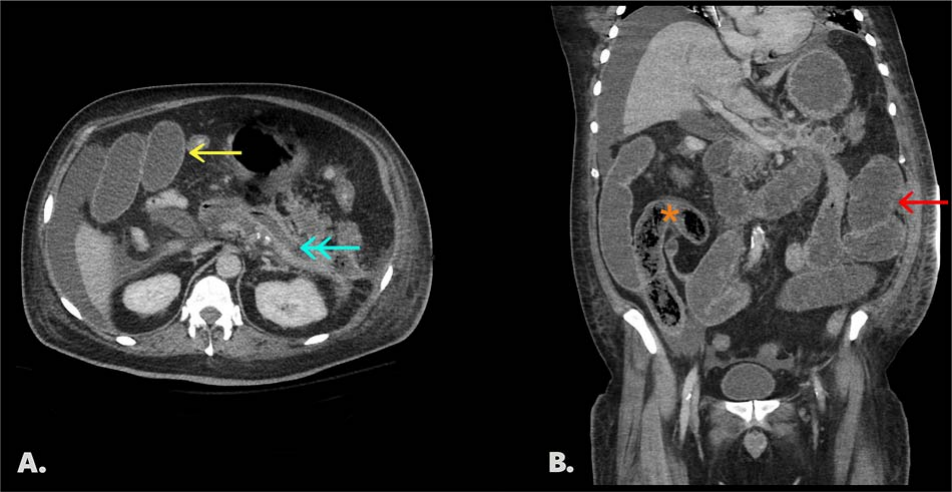
Follow-up CT with IV contrast (portal venous phase). Fig. 2A: an axial image shows a collapsed pancreatic bed (↞) containing trace fluid, dilated small bowel loops (←), and perihepatic free fluid. Fig. 2B: coronal image of same study shows interval migration of the bezoar into the distal small bowel (*) and upstream dilated, fluid-filled small bowel (←).

Final CT shows dilated fluid-filled small bowel loops with interval migration of the bezoar into the distal small bowel (Fig. 2).

**Figure 3:**
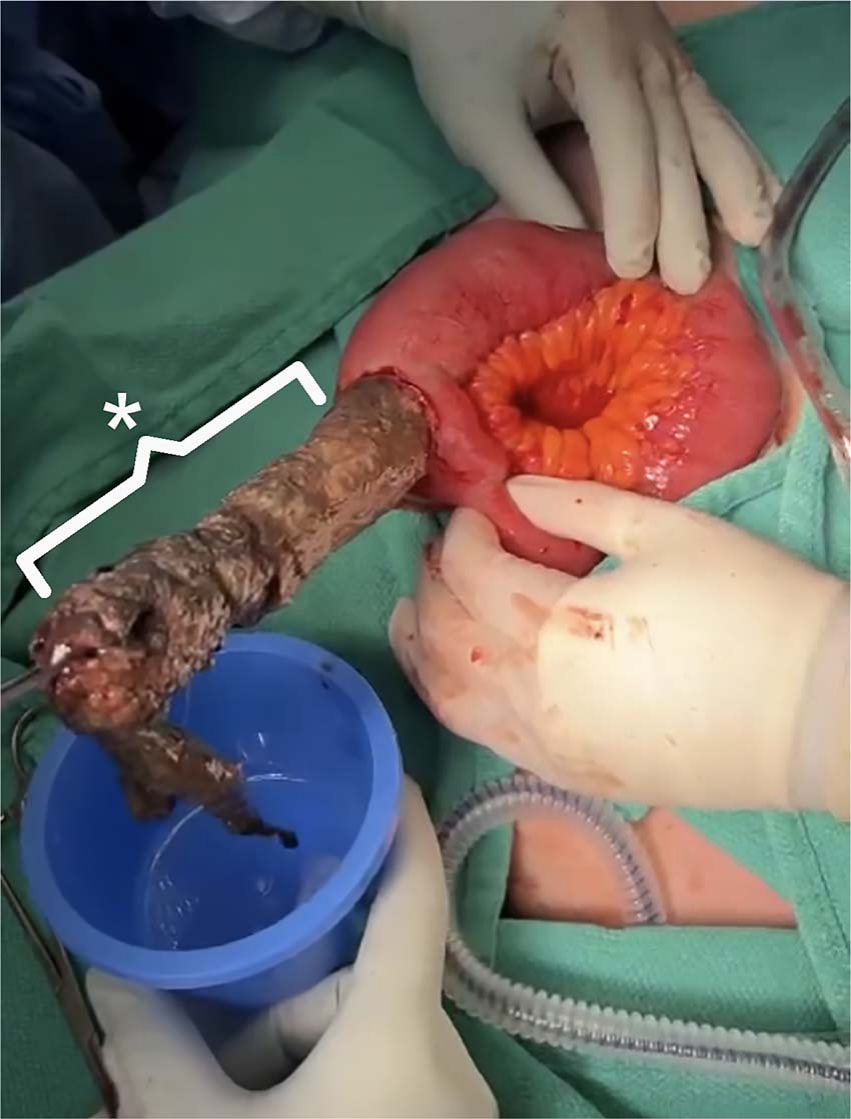
Intraoperative findings.

In the operating room, he underwent a diagnostic laparoscopy and was found to have a large mass within the terminal ileum, which was causing the obstruction. The small bowel was exteriorized through a midline incision, and a large bezoar was palpated within it. An enterotomy was performed to remove the bezoar of pancreatic necrosum (Fig. 3). The small bowel was closed, and the procedure was completed successfully.

The patient recovered from the operation, with restored gastrointestinal function shortly thereafter. Due to his complete pancreatic necrosis, he was discharged home on insulin and pancreatic enzymes.

## Discussion

Necrotizing pancreatitis is a severe form of pancreatitis characterized by necrosis of the pancreas and peripancreatic tissue. The incidence of pancreatic necrosis in patients with acute pancreatitis is ~10%–20% [1]. Historically, the management of infected pancreatic necrosis involved early surgical debridement of the pancreas. However, more recently, a step-up strategy beginning with endoscopic drainage followed by minimally invasive laparoscopic or retroperitoneal approaches to necrosectomy has emerged [4].

In this case report, we describe a patient who developed a spontaneous pancreaticoduodenal fistula secondary to necrotizing pancreatitis, with the resultant migration of pancreatic necrosum into the small bowel causing a mechanical obstruction. Only two cases have been previously reported where a bowel obstruction occurred secondary to a pancreas necrosum; however, in both of those cases the bezoar occurred after surgical cyst-jejunostomy [5, 6]. Our report, therefore, appears to be the first description of a bowel obstruction secondary to migration of pancreas necrosum through a spontaneous pancreaticoduodenal fistula. Importantly, in all reported cases, the pancreas bezoar migrated through a small bowel fistula. There have been no reports of transgastric pancreas necrosum migration, which is likely explained by ‘auto-debridement’ by gastric acid and enzymes’ digestion and liquefication of necrosum [7, 8]. Based on this, it would be expected that transgastric necrosectomy is less likely to result in necrosum migration. If drainage of pancreatic necrosum is performed through the duodenum or jejunum, we suggest the pancreatic bed should be cleared of all necrotic tissue to reduce this risk. Finally, early necrosectomy in patients with a spontaneous pancreaticoduodenal fistula may reduce the risk of spontaneous necrosum migration and, thus, morbidity and mortality in these patients.
